# Phonological and semantic strategies in a letter fluency task for people with Alzheimer’s disease

**DOI:** 10.3389/fpsyg.2022.1053272

**Published:** 2022-12-14

**Authors:** Jimin Park, Yae Rin Yoo, Yoonseob Lim, Jee Eun Sung

**Affiliations:** ^1^Department of Communication Disorders, Ewha Womans University, Seoul, Republic of Korea; ^2^The Center for Intelligent and Interactive Robotics, Korea Institute of Science and Technology, Seoul, Republic of Korea; ^3^Department of HY-KIST Bio-Convergence, Hanyang University, Seoul, Republic of Korea

**Keywords:** phonological strategy, semantic strategy, semantic relatedness, letter fluency, Alzheimer’s disease

## Abstract

**Objectives:**

This study investigated whether employing a phonological or semantic strategy elicited a better performance on a letter fluency task for people with Alzheimer’s disease (AD).

**Methods:**

Sixty participants with probable AD were extracted from the DementiaBank database. After applying exclusion criteria, 47 participants were included in the final analysis. We used phonological and semantic strategies to analyze participants’ responses to the letter fluency task. The phonological strategy analysis was based on the number of switches and the mean cluster size, and the semantic strategy analysis was based on semantic relatedness, which quantified word-similarity change by adapting the concept of persistence length from analyses of DNA and protein structures. We employed Pearson correlation coefficients to determine whether any strategy indexes were significantly related to the number of correct responses and used stepwise multiple regression analyses to determine the best predictor.

**Results:**

Participants who relied on phonological strategy performed better on the letter fluency task. The number of correct responses was significantly positively correlated with phonological strategy but significantly negatively correlated with semantic strategy. The number of switches, mean cluster size, and semantic relatedness were all significant predictors, explaining 68.1% of the variance.

**Conclusion:**

Our results suggested that individuals with AD who engaged in phonological strategy performed better on the letter fluency task than those who relied on semantic strategy.

## 1. Introduction

Dementia is a chronic or progressive syndrome that results in cognitive decline, affecting both memory and language (McKhann et al., 2011). According to previous studies, alterations in language may be an early indicator of cognitive decline (Forbes-McKay and Venneri, 2005; Ahmed et al., 2013), with untypical linguistic features acting as biomarkers for dementia (Fraser et al., 2015; Luz et al., 2018).

Verbal fluency tasks and other cognitive tasks constitute one of the commonly used methods for diagnosing neurological diseases, including Alzheimer’s disease (AD) in dementia (Tröster et al., 1998; Troyer et al., 1998; Gomez and White, 2006; Haugrud et al., 2011; Rofes et al., 2020). Category and letter fluency tasks, the two types of verbal fluency assessment, both assess an individual’s cognitive functions by giving specific constraints, such as time (e.g., 60 s) and task rules (e.g., specific semantic or phonemic categories). Researchers have postulated that verbal fluency tasks can be used to measure a wide variety of cognitive abilities such as executive functions, processing speed, lexical access, and memory (Braaten et al., 2006; Stolwyk et al., 2015; Aita et al., 2019). Given that multiple complex cognitive abilities are involved in performing verbal fluency tasks, verbal fluency abilities decline with age, along with naming abilities (Machado et al., 2018).

People with AD and mild cognitive impairment (MCI) perform worse in verbal fluency tasks than healthy older adults (Gomez and White, 2006; Haugrud et al., 2011; Weakley and Schmitter-Edgecombe, 2014; Mueller et al., 2015). While both category and letter fluency performance decline as AD progresses (Gomez and White, 2006), category fluency performance deteriorate more than letter fluency (Henry et al., 2004; Clark et al., 2009; Haugrud et al., 2011). Henry et al. (2004) performed a meta-analysis to examine the extent of the deficit between category and letter fluency tests. AD participants exhibited a greater mean effect size in the category fluency than in the letter fluency. Thus, most studies have focused more on category than letter fluency measures, given that semantic deficits become more salient than phonemic impairments even in the early stages of disease for individuals with MCI and AD (Henry et al., 2004; Shao et al., 2014).

However, the literature often conflicts, as some studies have indicated that people with AD perform worse on letter fluency tasks to a similar extent as on category fluency tasks (Suhr and Jones, 1998; Fisher et al., 2004). In terms of cognitive processing mechanisms associated with the two fluency tasks, category fluency tasks rely heavily on the semantic memory system, whereas letter fluency tasks tap into orthographic and phonemic relatedness, such as the construction of rhyming words (Baldo et al., 2006; Birn et al., 2010). Additionally, various neuroimaging studies have demonstrated that category and letter fluency tasks engage in diverse brain functional areas. Biesbroek et al. (2021) implemented structural disconnection and multivariate support vector regression to map lesion symptoms on verbal fluency tasks. They discovered that both type of tasks associates with the inferior frontal gyrus, but that the left temporal lobe was dissociated. In addition, the anterior lateral cortex is exclusively involved in letter fluency tests, and the medial and posterior cortex are exclusively involved in category fluency tasks. Nevertheless, different studies produce different findings about activities in brain areas between tasks (Baldo et al., 2006; Schmidt et al., 2017). Wagner et al. (2014) conducted a meta-analysis of brain activation on measures of verbal fluency. Except for the posterior-dorsal left inferior frontal gyrus, which was notably activated in the letter fluency task, they observed that category and letter fluency tasks did not result in distinct activation in brain areas.

Recently, letter fluency tasks have been conducted with diverse groups, such as healthy older adults, bilingual adults, those with aphasia, and those with age-related hearing loss (Kosmidis et al., 2004; Lanting et al., 2009; Faroqi-Shah and Milman, 2017; Bennett and Verney, 2018; Gonzalez-Burgos et al., 2020; Loughrey et al., 2020). However, few studies have been conducted on AD, even though AD exacerbates deficits in executive functions and cognitive decline which cause difficulties with letter fluency tasks. Braaten et al. (2006) realized that the results of letter fluency tests could help categorize different types of dementia. They demonstrated that individuals with vascular dementia performed significantly worse in the letter fluency tasks than those with frontotemporal dementia, although these differences were not observed in category fluency tasks. Previous studies have thus emphasized the importance of both letter fluency and category fluency tasks (Braaten et al., 2006; Gomez and White, 2006).

Traditional approaches to analyzing verbal fluency measures have focused on the number of accurate responses given in verbal fluency tasks. However, researchers have argued that qualitative analyses, including clusters and switching analyses, can provide more critical clinical insights and implications for understanding verbal fluency deficits in people with AD rather than only focusing on accuracy (Troyer et al., 1998; Gomez and White, 2006; March and Pattison, 2006; McDowd et al., 2011; Rofes et al., 2020).

Clustering and switching behaviors, word retrieval strategies used in verbal fluency tasks (Troyer et al., 1997; Weakley and Schmitter-Edgecombe, 2014), can measure cognitive flexibility (Troyer et al., 1997). Clusters comprise successfully generated words in the same semantic or phonological category, and switching refers to shifting between clusters (Troyer et al., 1997; Troyer, 2000). Previous research has reported that performing clustering and switching analyses is related to executive functions (Shao et al., 2014; Aita et al., 2019) because these behaviors require the capacity to uphold the task’s objective while simultaneously updating and shifting the categories (Miyake et al., 2000; Shao et al., 2014; Rofes et al., 2020). Therefore, participants need to keep track of their spoken words by engaging a clustering strategy within a given category and must employ the switching strategy when their semantic or phonemic exemplars start running out of lexical or phonological storage. Qualitative analyses have gained considerable attention by capturing more sensitive features of deficits in AD, as they can tap into cognitively demanding components of verbal fluency measures.

Research on switching and clustering analyses has been increasingly conducted on verbal fluency measures. However, most studies have concentrated on category fluency measures (Raoux et al., 2008; Haugrud et al., 2011; Rofes et al., 2020), with relatively few studies conducted on letter fluency analyses for AD (Gomez and White, 2006; Haugrud et al., 2011; McDowd et al., 2011).

Troyer et al. (1998) studied 23 people with AD, 11 people with dementia and Parkinson’s disease (DPD), 11 people with non-dementia Parkinson’s disease (NPD), and 38 controls. Their results showed that AD sufferers generated significantly fewer words and clusters than the control group in both category and letter fluency tasks. However, AD sufferers were only impaired in the category fluency task, indicating that lexical-phonemic memory remains relatively intact. This result is consistent with studies by Tröster et al. (1998) and Haugrud et al. (2011). Tröster et al. (1998) conducted category and letter fluency tasks in four groups (controls, AD, DPD, and NPD) of 30 participants. In the category fluency task, they found that only AD patients made significantly fewer switches and that the proportion of clusters and switches was highly correlated with disease severity. Haugrud et al. (2011) compared 26 controls and 26 individuals with AD. They determined that while people with AD generated significantly fewer correct words and novel clusters than the control group, both groups produced a similar number of switches and clusters and their respective mean cluster sizes did not differ significantly in the letter fluency task. However, these previous studies cannot be extrapolated to interpret the linguistic deficits of AD because their sample sizes are too small.

The current study analyzes letter fluency performance in people with AD by employing qualitative analysis approaches and comparing phonological and semantic strategies. Analyses of clusters and switching from Troyer’s (2000) criteria are classified as phonological strategies, and the analysis of semantic relatedness between words is classified as a semantic strategy. In addition, we investigate which strategy best predicts the correct number of words in the letter fluency task. As no study has yet analyzed phonological and semantic strategies in letter fluency measures with AD to our knowledge, this study proposes a new method of analyzing letter fluency tasks in AD. To this end, we hypothesize that implementing the phonological strategy will facilitate the correct responses to the letter fluency task.

## 2. Methods

### 2.1. Participants

Participants with probable AD (*n* = 60) were selected from the Pittsburgh corpus of DementiaBank (Becker et al., 1994) – an open-access database supported by NIH-NIDCD grant R01-DC008524. Thirteen participants were excluded from the original corpus because (1) one participant with an irrelevant audio file, (2) nine participants generated less than four words (the minimum requirement for semantic strategy analysis), and (3) three participants were identified as outliers on the outcome measures (>3SD above average; Qiao et al., 2021). The final statistical analysis included 47 total participants. The average age of the participants was 72.28 years (SD = 8.60, Range = 56–88), with an education average of 11.79 years (SD = 2.66, Range = 6–20). The average score for the Mini-Mental State Examination (MMSE; Folstein et al., 1975) was 19.11 (SD = 3.88, Range = 10–26).

### 2.2. Task and analysis

We analyzed a letter fluency task where participants were given 60 s to say as many words as possible starting with the letter *f*. Participants’ responses were analyzed using phonological and semantic strategies.

#### 2.2.1. Phonological strategy analysis

Based on Troyer’s (2000) criteria, the number of switches and mean cluster size were calculated for each participant. Clusters were defined as successively generated words that adhered to the following criteria: words beginning with the same first two phonemes (e.g., find-fire), words that rhymed (e.g., fry-fly), words differing only by a vowel sound (e.g., flip-flop), and homonyms (e.g., fair-fare; Troyer et al., 1997; Troyer, 2000). The second word in each cluster evaluated cluster size; thus, a single word was calculated as a cluster size of 0 and two words as a cluster size of 1. The mean cluster size was calculated by dividing the total cluster size by the number of clusters employed by each participant. Transitions between clusters were counted as switches. Errors and repetitions were included in clusters and switches to indicate the cognitive processes employed by the participants (Troyer et al., 1997; Troyer, 2000). However, they were excluded when counting the number of correct words.

We classified clusters and switches as a phonological strategy index. Two doctoral students and four master’s students majoring in communication disorders evaluated the two measures. The raters were trained using Troyer’s (2000) criteria and practiced using the scoring system until they reached 100% agreement.

#### 2.2.2. Semantic strategy analysis

For the semantic strategy, we used “semantic relatedness” as an index by evaluating the degree of change in the semantic similarity of word pairs for each participant. We used spaCy – a leading open-source Python library for natural language processing – to represent a word as a vector and calculate the cosine angle between each word pair based on the order generated by the participants. The cosine angle between two-word vectors represents words’ degree of semantic similarity. For instance, when a word and the following word are semantically similar, the cosine angle between the vectors of two words is close to 0°. In contrast, the cosine angle approaches ±180° when two words are semantically dissimilar.

**Figure 1:**
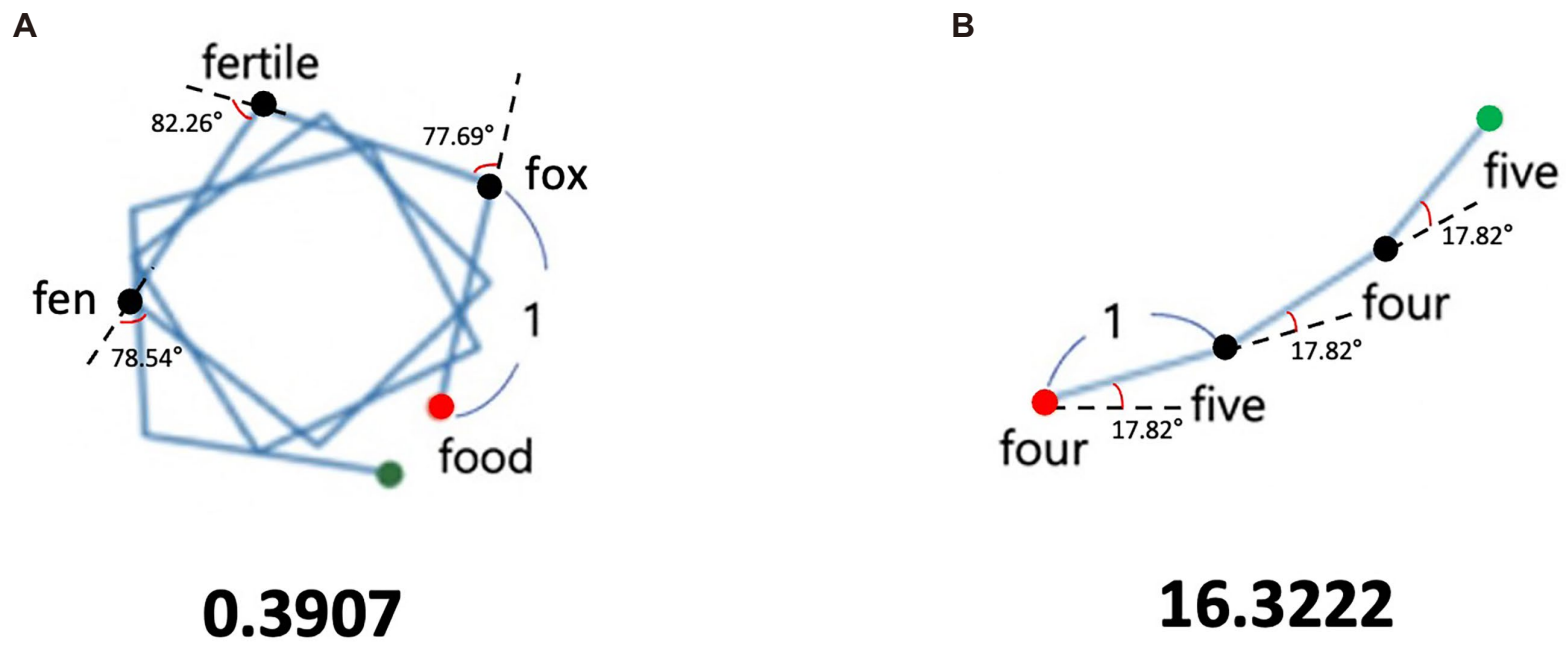
Example of semantic relatedness for low and high cases. **(A)** Low value of semantic relatedness. **(B)** High value of semantic relatedness. The red dot is the starting point, and the green dot is the end point.

Then, as shown in Figure 1, we constructed a diagram with discrete lines where the angle between lines corresponds to the cosine angle. When spoken words are semantically similar, such as “four-five-four-five,” the cosine angles between the word vectors become “17.82° – 17.82° – 17.82°,” and the graph forms a less curved line (Figure 1B). However, when spoken words are semantically disassociated, such as “food – fox – fertile – fen,” the cosine angles between the word vectors become “77.69° – 82.26° – 78.54°,” resulting in a graph with a more bent shape (Figure 1A). This shows that a graph’s curvature could represent the degree of change in semantic similarity and the semantic relatedness in a chain of words produced by the participant. To quantify graph curvature, we applied the concept called “persistence length,” which is often used to analyze the bending rigidity of a DNA chain or a polymer (Flory, 1989; Rubinstein and Colby, 2008). As shown at the bottom of Figure 1, a sequence of less semantically related words has a shorter persistence length than that of more semantically related words. Participants who produced less than four words were not included in the analysis as semantic relatedness of a graph could not be accurately estimated with only one or two cosine angles.

### 2.3. Statistical analyses

Three participants were excluded before the statistical analyses because they exceeded the outlier threshold (>3SD above average; Qiao et al., 2021). One participant was identified as an outlier in the mean cluster size, while the other two were identified from semantic relatedness. The graph of the excluded participant with the highest value in semantic relatedness is Figure 1B.

We calculated Pearson correlation coefficients to determine which strategy most correlates with the number of correct words in the letter fluency task. We also conducted stepwise multiple regression analyses to identify the best predictor for the number of correct words in the letter fluency task. All statistical analyses were performed using IBM SPSS Statistics (Statistics Package for the Social Science, version 28.0) for Windows, and statistical significance was defined at the significance level of 0.05.

## 3. Results

### 3.1. Descriptive statistics of phonological and semantic analyses

**Figure 2:**
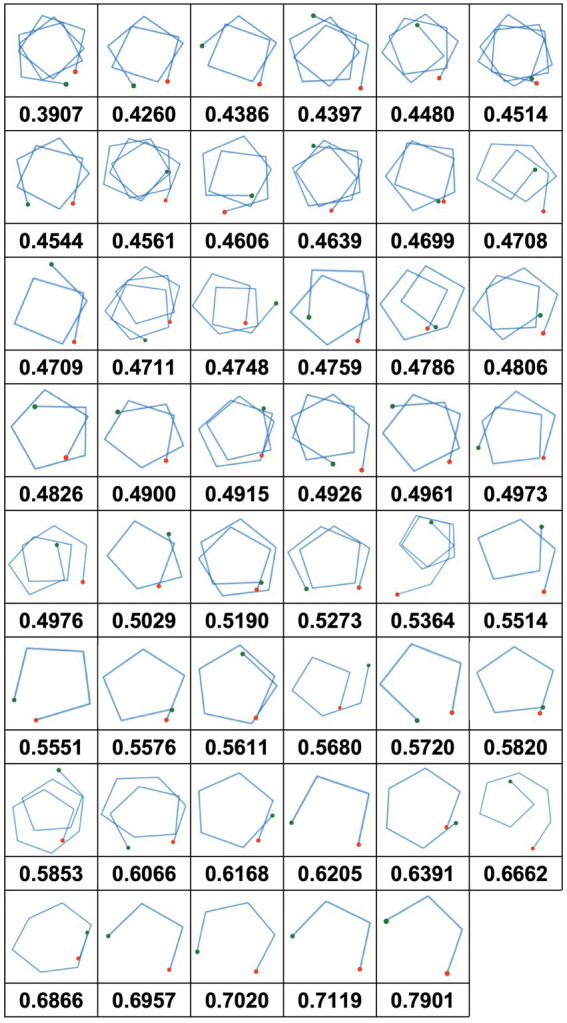
Individual plots of semantic relatedness values for each participant. Plots are provided in ascending order according to semantic relatedness values. The red dot in each plot represents the starting point, and the green dot represents the end point.

Table 1 shows descriptive statistics for each phonological and semantic strategy measure and the number of correct words. Phonological strategy includes mean cluster size and the number of switches as an index, while semantic strategy includes semantic relatedness. Individual plots of semantic relatedness for each participant are shown in Figure 2.

**Table 1:** Descriptive statistics of phonological and semantic strategy measures.

	NoCW	Phonological strategy		Semantic strategy
		MCS	NoS	SR
Mean (*SD*)	6.87 (3.24)	0.15 (0.17)	7.09 (2.87)	0.53 (0.09)
Range	1–15	0–0.66	2–14	0.39–0.79

NoCW, number of correct words; MCS, mean cluster size; NoS, number of switches; SR, semantic relatedness; SD, standard deviation.

### 3.2. Analyses of Pearson correlation coefficients

We calculated the Pearson correlation coefficients to determine if there were any significant relationships between number of correct words, phonological strategy measures (mean cluster size, number of switches), the semantic strategy measure (semantic relatedness), and demographic factors (age, education, MMSE scores). The number of correct words was significantly positively correlated with the number of switches (*r* = 0.661, *p* < 0.001), followed by the mean cluster size (*r* = 0.328, *p* = 0.024) and MMSE scores (*r* = 0.350, *p* = 0.016). In contrast, semantic relatedness was significantly negatively correlated with the number of correct words (*r* = −0.721, *p* < 0.001), indicating that participants with high semantic relatedness values tended to produce fewer correct words in the letter fluency task. Semantic relatedness was also significantly negatively correlated with the number of switches (*r* = −0.523, *p* < 0.001) and the mean cluster size (*r* = −0.344, *p* = 0.018). The correlational results are shown in Table 2.

**Table 2:** Results of Pearson correlation coefficients.

	Phonological strategy		Semantic strategy	Demographic factors		
	MCS	NoS	SR	Age	Edu	MMSE
NoCW	0.328^*^	0.661^***^	−0.721^***^	−0.091	0.012	0.350^*^
MCS	1	−0.114	−0.344^*^	0.089	0.165	0.133
NoS	–	1	−0.523^***^	−0.067	0.142	0.266
SR	–	–	1	−0.035	0.026	−0.23

NoCW, number of correct words; MCS, mean cluster size; NoS, number of switches; SR, semantic relatedness; Edu, education; MMSE, mini-mental state examination (Folstein et al., 1975). ^***^p < 0.001, ^**^p < 0.01, ^*^p < 0.05.

### 3.3. Analyses of stepwise multiple regression

We conducted a stepwise multiple regression analysis to examine the predictors for the number of correct words from the letter fluency task. Our independent variables were the mean cluster size, number of switches, semantic relatedness, age, education, and MMSE. We tested continuous variables for multicollinearity and showed that it was not an issue (mean cluster size, tolerance = 0.763, VIF = 1.311; number of switches, tolerance = 0.629, VIF = 1.590, semantic relatedness, tolerance = 0.562, VIF = 1.780). In the final model for the number of correct words, the number of switches (*β* = 0.558, *p* < 0.001), the semantic relatedness (*β* = −13.765, *p* = 0.002), and the mean cluster size (*β* = 5.124, *p* = 0.013) were all significant predictors [*F*(3,46) = 30.550, *p* < 0.001, *R*^2^ = 0.681] of the correct number of words. Together, they explained 68.1% of the variance in our independent variables.

## 4. Discussion

Our study conducted qualitative analyses on the letter fluency measures of people with AD by scrutinizing phonological and semantic strategies. We employed well-known methods to analyze phonological strategies such as mean cluster size and the number of switches. However, we used a new method to analyze semantic strategy using semantic relatedness. We analyzed these strategies to examine what strategies people with AD can employ to perform well on the letter fluency task. Few studies have reported the letter fluency performance of AD sufferers by analyzing the mean cluster size and the number of switches with the number of correct responses (Tröster et al., 1998; Troyer et al., 1998; Haugrud et al., 2011). However, not many studies have analyzed letter fluency as opposed to category fluency tasks in people with AD. This is likely due to the assumption that the neurodegenerative process starts in the semantic domains rather than the phonemic components (Mickes et al., 2007). When people are asked to produce words starting with a specific phoneme, they will likely start searching through their verbal lexicon by activating the given phoneme. However, once it is phonologically activated by combining the given phoneme with vowels, the whole syllable is not independent of lexical representations associated with it (Cutler et al., 1987; Christophe et al., 2004). Considering this process, the current study analyzes how people with AD rely either on phonological or semantic strategies and how these strategies account for performance regarding the number of correct responses.

Our analysis of the phonological strategy revealed that both the number of switches and the mean cluster size were significantly positively correlated with the number of correct responses. This indicates that AD sufferers with a larger mean cluster size and frequent switching behaviors performed better on the letter fluency task. These results are in line with previous reports that have suggested that switching performance in AD is a significant predictor of the number of correct responses in the letter fluency task (Gomez and White, 2006; McDowd et al., 2011). In addition, Gomez and White (2006) conducted letter and category fluency measures on a healthy control group and people with mild AD. Their results indicated that the number of correct responses in both letter and category fluency tasks was significantly positively correlated with the number of switches, the number of clusters, and the mean cluster size, which was consistent with our results on the letter fluency measure.

Several studies have examined how the qualitative analyses of fluency measures are related to the number of correct responses from healthy older adults (Troyer et al., 1997; Robert et al., 1998; Kosmidis et al., 2004) and those suffering from neurodegenerative diseases such as MCI (Mueller et al., 2015) and Parkinson’s disease (PD; Galtier et al., 2017). For example, Kosmidis et al. (2004) assessed the letter and category fluency performance of 300 healthy Greek adults of various ages, with an average age of 46 years old. They found that switching and clustering behaviors significantly correlated with the number of correct responses on both fluency tasks.

Troyer et al. (1997) also concluded that the number of switches correlates significantly with the number of correct responses in both letter and category fluency measures in younger (*n* = 41) and older (*n* = 54) Canadian participants. Similar findings were reported in people with MCI (Mueller et al., 2015) and PD (Galtier et al., 2017), which respective studies reported that individuals with amnestic MCI or PD generated significantly more correct responses in both category and letter fluency tasks, as they displayed more frequent switching behaviors. These findings were consistent across different neurodegenerative groups, including healthy adults, and support the hypothesis that switching, and clustering behaviors are related to the function of the frontal lobe (Abwender et al., 2001), especially to its executive functions (Alvarez and Emory, 2006).

Additionally, March and Pattison (2006) analyzed category fluency tasks performed by people with AD and healthy older adults. They reported that the total number of words in the “animal” category significantly correlated with the number of switches and subcategories in both groups. Rofes et al. (2020) demonstrated similar results by computing conditional inference trees in a random forest in the animal fluency task on people with AD. Their results indicated that participants who produced more than 5.8 switches generated more correct words than those who produced fewer than 5.8 switches. Therefore, our current study complements previous studies which have suggested that the number of correct responses significantly correlates with the number of switches on letter and category fluency measures in people with AD.

To analyze semantic strategy, our study employed semantic relatedness. Very few studies have examined strategic components regarding which semantic and phonological components contribute to overall performance in AD. Raskin et al. (1992) conducted a similar study using strategic analysis for both category and letter fluency tasks in people with non-dementia PD. They analyzed semantic and phonemic cluster ratios for each letter task (letter fluency task with a letter *f*) and a category task with an “animal” category. Their results showed that more phonemic cluster ratios were elicited on the letter task than semantic clusters. In contrast, significantly greater semantic cluster ratios were produced than phonemic cluster ratios on the category fluency task. Their results support findings in the current study that phonological clusters and switching behaviors significantly positively correlate with performance on the letter fluency task. However, as indexed by semantic relatedness, semantic strategy was significantly negatively correlated with overall performance on the letter fluency task.

Vonberg et al. (2014) also analyzed semantic and phonological search strategies in the letter fluency task (using the letter *s*) in healthy German adults. Their results were comparable to ours in that they indicated a significantly positive correlation between phonemic relatedness and the number of correct words. However, their study demonstrated a weak negative correlation between the number of correct words and semantic relatedness. Their results suggested that the phonological search activates words starting with a typical letter, whereas employing semantic search strategies acts as an obstacle for letter fluency measures, which is in line with the current study. Furthermore, Vonberg et al. (2014) analyzed the semantic strategy on the letter fluency task using a rating scale (0–4) for semantic relatedness surveyed by non-experts. However, our methodology for analyzing semantic strategy differed from theirs because our methodology objectively represented semantic strategy by quantifying semantic similarity using the corpus-based semantic space, spaCy.

In conclusion, we conducted a qualitative analysis of the number of switches and mean cluster size for the phonological strategy and the semantic relatedness for the semantic strategy in the letter fluency task. Our findings indicate that generating words using the phonological strategy rather than the semantic strategy increases accuracy during the letter fluency task. Our study is novel in that we attempted to quantify word retrieval patterns, particularly those involved in semantic activation, using an open-source corpus such as spaCy to objectively represent semantic strategies. Moreover, this study provided evidence on the letter fluency performance of people with AD, because there has been relatively little research on letter fluency measures compared to category fluency tasks. However, this study has limitations because we only analyzed performance of letter fluency tasks. Therefore, further studies are necessary to explore semantic and phonological strategies in category and letter fluency tasks by applying this study’s approach.

## Data availability statement

Publicly available datasets were analyzed in this study. This data can be found at: DementiaBank, https://dementia.talkbank.org/.

## Ethics statement

Ethical review and approval was not required for the study on human participants in accordance with the local legislation and institutional requirements. Written informed consent for participation was not required for this study in accordance with the national legislation and the institutional requirements.

## Author contributions

JP analyzed the data, carried out the statistical analysis, and wrote the manuscript. YY analyzed the data and assisted with writing the manuscript. YL contributed to the data analysis and also assisted with writing the manuscript. JS formulated the research design and supervised the whole research process. All authors contributed to the article and approved the submitted version.

## Funding

This research was partly supported by the National Research Council of Science & Technology (NST) grant by the Korea Government (MSIT) (No. CAP21052-000), the National Research Foundation of Korea (NRF) grant funded by the Korea Government (MSIT) (2022R1A2C2005062), and Basic Science Research Program through the National Research Foundation of Korea (NRF) funded by the Ministry of Education (NRF-2022R1I1A4063209).

## Conflict of interest

The authors declare that the research was conducted in the absence of any commercial or financial relationships that could be construed as a potential conflict of interest.

## Publisher’s note

All claims expressed in this article are solely those of the authors and do not necessarily represent those of their affiliated organizations, or those of the publisher, the editors and the reviewers. Any product that may be evaluated in this article, or claim that may be made by its manufacturer, is not guaranteed or endorsed by the publisher.
